# Complete mitochondrial genome sequence of *Catla catla* (Hamilton, 1822) from the Halda river of Bangladesh

**DOI:** 10.1080/23802359.2020.1809542

**Published:** 2020-08-30

**Authors:** M. M. Kibria, N. Islam, M. Billah, K. S. M. Shawrob, M. H. Rumi, AMAM Zonaed Siddiki

**Affiliations:** a Department of Zoology, University of Chittagong, Chittagong, Bangladesh; b Halda River Research Laboratory, University of Chittagong, Chittagong,Bangladesh; c Genomics Research Group, Chittagong Veterinary and Animal Sciences University (CVASU), Chittagong, Bangladesh; d College of Animal Science and Technology, Northwest A&F University, Yangling, China; e Department of Biotechnology, Inland Norway University of Applied Sciences, Elverum, Norway

**Keywords:** *Catla catla*, mitochondrial genome, protein-coding gene, tRNA, rRNA

## Abstract

Catla (*Catla catla*) is one of the fastest-growing major carp found in South Asia as well as Bangladesh. *Catla catla* is the second most popular indigenous carp species in the freshwater aquaculture industry of Bangladesh due to its relatively good taste and high market price. In this study, we disclosed the complete mitochondrial genome sequence of Bangladeshi Catla fish from Halda river located in Chittagong. The circular mitogenome of *Catla catla* is 16,597 bp in length and nucleotide composition is AT-based (72%), contains 37 genes including 13 protein-coding genes, 22 tRNA genes, 2 rRNA genes and a D-loop (control region).

## Introduction

*Catla catla* is a member of the Cyprinidae family, which is endemic to the perennial river network of northern India, the Indus plain and adjacent hills of Pakistan, Bangladesh, Nepal and Myanmar (Reddy 1999). It has become one of the most well-established fish populations of all the rivers, lakes and reservoirs where they have been introduced. The Halda River is located in South-East region of Bangladesh which is a major tributary of the river Karnaphuli in Chittagong district originated from the hilly Haldachora fountain at the Patachara hill ranges of Ramgarh in the Khagrachari hill and renowned for being the only natural spawning ground of Indian major carp in Bangladesh (Tsai et al. 1981; Akter and Ali 2012; Kabir et al. 2015). A major portion of the country’s pond carp culture is dependent on these wild seed that has an important and potential contribution in the agro-based economic development, poverty alleviation, employment, supplying of animal protein and earning the foreign currency for the national sector (Azadi 1979, DoF 2005). *C. Catla* is one of the "Four famous Indian carp" of Halda river which has extensive demand in carp polyculture system among the fish farmers due to it's higher productivity rate and compatibility with other major carps, specific surface feeding habit that help increase water quality, enriched protein and vitamin content with lower calories, delicate flavor and consumer preference (Shafi and Quddus, 1982). For being small in size, high evolutionary rate, and maternal inheritance mood, the complete mitochondrial genome sequences provide insight into the assessment of wide variation in animals and the comparison of sequence data contribute to the exploration of improved markers for population ecological studies (Avise 1995; Zhou et al. 2009). Here we reported the entire mtDNA sequences of Catla* catla* from the Halda river.

The specimen was collected from Halda river, Chittagong (geographic coordinate: 22°33′34.7″ N 91°50′41.8″E). Fresh tissue (from muscle) sample was stored at −20 °C until used to isolate genomic DNA using commercial DNA extraction kit (AddBio, Korea) and the total DNA was stored with a voucher number (DPP/CVASU/2019-12-44). Purified DNA was sent for library preparation and sequencing through commercial suppliers. DNA was sequenced using Illumina NovaSeq 6000 platform from BGI, China. The mitochondrial genome reads were separated from the whole genome sequence by mapping it against the reference Catla mitochondrial datasets (KY419138) using SAMTOOLS. The organelle assembler NOVOPlasty V.2.7.2 (Dierckxsens et al. 2017) was used to assemble the clean reads. Web-based tools like MITOS (Bernt et al. 2013) and GeSeq (Tillich et al. 2017) were applied to perform structural and functional annotation. Another tool, OGDRAW was used to construct the circular representation of the entire mitogenome (Greiner et al. 2019). Finally, mtDNA sequences were aligned and a phylogenetic tree was constructed by using CLC Main Workbench.

The complete mitogenome of *Catla catla* (NCBI accession number **MT303069**) is 16,597 bp in length and consists of 13 protein-coding genes, two ribosomal RNA genes (rRNA), 22 transfer RNA (tRNA) genes, and a putative control region (D-loop). The structural organization and location of the different features of these mitogenomes were consistent with the common vertebrate mt genome model (Liu and Cui 2009). The relative order of nucleotide composition corresponds to the nucleotide pattern of other fish mitogenomes *A* > *C*>*T* > *G* (Wang et al. 2008). The mitochondrial genome of *Catla catla* contains an *A* + T bias with an overall nucleotide composition of *A* = 5383 (32.43%), *T* =4087(24.62%), *C* = 4580 (27.60%), and *G* =2547(15.35%). The *GC* content of the mitogenome is 42.94 %. Furthermore, the AT-skew is positive which is 0.13 and *GC*-skew is observed negative which is −0.28.

**Figure 1.**
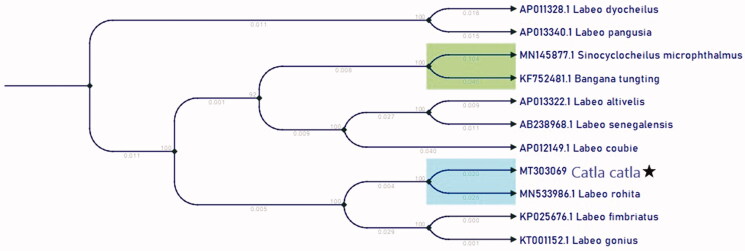
The neighbour-joining tree of *Catla catla* and 9 Labeo based on the complete mitochondrial genome. Numbers above the branches indicate the bootstrap support values, and values lower than 50 are not shown.

Most of the protein-coding genes (PCGs) have been encoded on the H-strand of mtDNA. Only one PCG (*nad6*) and 8 transfer RNA genes (*trnA, trnC, trnE, trnN, trnP, trnS2, trnY*) were encoded in the L-strand of mtDNA. Most of the PCG starts with a standard ATG start codon, whereby *nad2, nad1, nad5,* starts with ATA and *Cox2* starts with AAT. The length of the 12S rRNA and 16S rRNA genes were 952 bp and 1685 bp respectively. The tRNA genes encoded in the genome ranged from 60 to 75 bp. The control region is between *trnaP* and *tnaF* and has a size of 930 bp. The structural organization and gene order of mtDNA sequence of *Catla catla* correspond to other common carp strains (Chang et al. 1994; Wang et al. 2013; Liu et al. 2016, Ye et al. 2018). The phylogenetic relationship was estimated using neighbour-joining method implemented in CLC main workbench (Fig. 1). Two different closely related species, *Catla catla* and *Labeo rohita* were placed in the sister clade. All other *Labeo* were also placed in different sister clades.

In a nutshell, this study provides the information of *Catla catla* mitogenome collected from the Halda river of Bangladesh. Thus, the data presented here will be a valuable resource for future research by the fish geneticists and evolutionary biologists for improving the aquaculturally important traits of indigenous fish - *C. catla* and other key cyprinid species. This study will also provide crucial information for further taxonomic and phylogenetic analyses among closely related species and implementation of the effective conservation strategy by establishing a live gene bank to supply pure strains of this unique resource of Bangladesh.

## Data Availability

The data that support the findings of this study are openly available in NCBI Nucleotide database(https://www.ncbi.nlm.nih.gov/nucleotide/) under the accession number MT303069
